# Attraction of *Anopheles gambiae *to odour baits augmented with heat and moisture

**DOI:** 10.1186/1475-2875-9-6

**Published:** 2010-01-06

**Authors:** Evelyn A Olanga, Michael N Okal, Phoebe A Mbadi, Elizabeth D Kokwaro, Wolfgang R Mukabana

**Affiliations:** 1 International Centre of Insect Physiology and Ecology, PO Box 30772 - 00100, GPO, Nairobi, Kenya; 2 Department of Zoological Sciences, Kenyatta University, PO Box 43844 - 00100 GPO, Nairobi, Kenya; 3 School of Biological Sciences, University of Nairobi, PO Box 30197 - 00100 GPO, Nairobi, Kenya

## Abstract

**Background:**

The search for a standard human surrogate in the form of a synthetic mosquito attractant has been the goal of many laboratories around the world. Besides alleviating the occupational risk subjected to volunteers participating in vector surveillance and control, discovery of potent attractants underpins the development and deployment of mass trapping devices for controlling mosquito-borne diseases.

**Methods:**

A dual-port olfactometer was used to assess behavioural responses of female *Anopheles gambiae *mosquitoes towards synthetic versus natural (whole human emanations and worn socks) attractants. The synthetic attractants included a standard blend consisting of ammonia, carbon dioxide and water; and Ifakara blend 1 (IB1) consisting of various aliphatic carboxylic acids. Natural attractants were obtained from two males known to be less and highly attractive (LA and HA, respectively) to the mosquitoes. Mosquito responses to the volunteers' worn socks were also investigated. The effect of heat (25-27°C) and moisture (75-85%) on the mosquito behavioural responses was determined.

**Results:**

A significantly higher proportion of mosquitoes was attracted to each volunteer when compared to the standard blend. Whereas the proportion of mosquitoes attracted to person LA versus IB1 (49% versus 51%, respectively; P = 0.417) or his worn socks did not differ (61% versus 39%, respectively; P = 0.163), far more mosquitoes were attracted to person HA relative to IB1 (96% versus 4%; P = 0.001) or his worn socks (91% versus 9%; P = 0.001). Person HA attracted a significantly higher proportion of mosquitoes than his worn socks, the standard blend and IB1 when these were augmented with heat, moisture or both (P = 0.001). Similar results were obtained with person LA except that the proportion of mosquitoes attracted to him versus his worn sock augmented with heat (P = 0.65) or IB1 augmented with heat and moisture (P = 0.416) did not differ significantly.

**Conclusions:**

These findings indicate that olfactory cues are key mediators of the mosquito host-seeking process and that heat and moisture play a minor role. The need for a standard, highly stringent positive control for screening synthetic attractants is strongly highlighted.

## Background

*Anopheles gambiae sensu stricto*, i.e. the principal vector of malaria in sub-Saharan Africa [1-4], locates its blood meal hosts largely based on olfactory cues [5]. Physical cues, encompassing heat and moisture, also play a role that is hitherto not well understood [6]. Dissecting and analysing the broad spectrum of human emanations [7] can provide an important basis for developing synthetic compounds or blends with desirable attractant [8], repellent or attractant "masking" properties [9]. Certainly, several compounds identified from human emanations have been demonstrated to exhibit attractant properties under varying experimental conditions [10-15].

Although the objectives of developing potent insect attractants are diverse, the central goal lies in pest and vector control [8,16]. With respect to mosquitoes, impact on target populations can be achieved by mass trapping [17,18] or lure and kill technology [19]. Despite the underlying conceptual, technical, logistical and financial limitations, mass trapping has been most explored for mosquito population reduction [16]. These strategies are, regardless of few field successes [17,18], still being developed. Current efforts centre on searching for new attractants and attractant formulations [14,15,20,21], improving on existing ones [13], and developing trapping devices [22,23]. Efficacy trials under field [14] and semi-field conditions are also underway [15,24].

The potency of an attractant is determined by comparing the relative attraction of target insects to it, versus its natural source or calculating the percentage of catch from a known insect population [16,25]. Thus, candidate synthetic attractants of anthropophilic mosquitoes have been evaluated by comparing their attractiveness to host-seeking mosquitoes against odour from human feet [24-27] and hands [28-30]. No attempts have been made to evaluate the efficacy of candidate attractants based on the full spectrum of human body emanations, yet this is what determines the end point of the entire host-seeking process [31]. Identifying a synthetic blend that attracts mosquitoes much the same as a human being [32] can enhance its usefulness in developing powerful tools for vector surveillance [5] and control [8,16,19].

In this study, the behavioural responses of *An. gambiae *towards two candidate synthetic attractant blends were evaluated. The key objectives were to (i) determine the relative response of *An. gambiae *towards the attractants in comparison to human volunteers, and (ii) investigate the effect of heat and moisture on mosquito responses to the synthetic attractants. Attraction of mosquitoes to the blends used in this study had been determined previously [15].

## Methods

### Mosquitoes

Experiments were conducted using the Mbita strain of *Anopheles gambiae sensu stricto *(hereafter referred to as *An. gambiae*). The mosquitoes were reared under ambient climatic conditions at insectaries of the International Centre of Insect Physiology and Ecology (ICIPE) located at Mbita Point, western Kenya. Mosquito eggs were dispensed into plastic trays containing filtered water from Lake Victoria. Hatched larvae were fed on Tetramin^® ^baby fish food three times per day. Pupae were harvested daily and transferred into clean cups half-filled with filtered lake water. Cups containing pupae were placed in mesh-covered cages (30 × 30 × 30 cm) prior to adult emergence. Emerged adult mosquitoes were fed on 6% glucose solution through wicks made from adsorbent tissue paper. All experiments utilized 100 adult female mosquitoes aged 3-6 days old. The mosquitoes were starved for 8 hours and did not receive a blood meal before experiments were commenced. Starving mosquitoes were provided with water on cotton towels placed on top of mosquito holding cups.

### Behavioural stimuli

Two male volunteers, aged 32 and 33 years old, acted as natural sources of host-seeking cues. One of the volunteers was less attractive (LA) and the other highly attractive (HA) to host-seeking, female *An. gambiae *mosquitoes [33]. The volunteers' wore cotton socks for 8 hours to collect their foot odours. This acted as an alternative natural source of host-seeking cues. The volunteers bathed with non-perfumed soap half an hour before onset of experiments. They wore short trousers only during the experimental periods. Their malaria parasite infection status was determined daily through microscopic examination of giemsa stained blood smears.

Synthetic stimuli were derived from two odour blends i.e. a standard blend [consisting of CO_2 _(500 ml/min), ammonia (2.5%) and distilled H_2_O] and Ifakara blend 1 (IB1) [consisting of propionic acid (0.1%), butanoic acid (1%), pentanoic acid (0.01%), 3-methyl butanoic acid (0.001%), heptanoic acid (0.01%), octanoic acid (0.01%), tetradecanoic acid (0.01%), ammonia (2.5%), lactic acid (85%), distilled water and carbon dioxide (500 ml/min)]. Strips of nylon sock material (90% polyamide and 10% spandex) measuring 26 cm long by 1 cm wide were dipped in separate one millilitre volumes of chemical constituents characteristic of a specific blend. Individual strips were not dipped in more than one chemical. Soaked strips were air dried at room temperature for five hours prior to experiments [15]. To constitute a blend a number of dried strips, selected based on the components of the standard blend or blend IB1, were tied together on one end prior to use as bait in experiments. The carbon dioxide component of the blends was supplied through gas tubing. All chemicals, except CO_2_, were purchased from Sigma-Aldrich Chemie GmbH (Germany). Carbon dioxide (industrial grade) was purchased from Carbacid Investments Ltd, Kenya.

### Experimental setup

**Figure 1:**
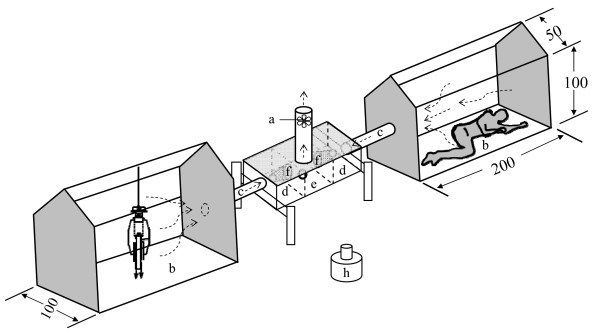
**The dual-choice experimental setup**. The fan (a) drew air (~130 L/min/tent) from the two tents (b) to the outside environment via PVC pipes (c), trap chambers (d) and central choice chamber (e). An exit trap (f) opened into each trap chamber. The fan pipe and the release cup (h) were fitted on top and on the bottom of the choice chamber, respectively, through circular holes. The trap and choice chamber measured 30 × 15 × 20 and 30 × 20 × 20, respectively. Diagrams are not drawn to scale; all dimensions are in centimeters.

All experiments were carried out using a model of a previously described olfactometer [33] modified to accommodate two, instead of three human subjects as sources of host-seeking stimuli (Figure 1).

### Testing functional integrity of the dual-choice setup

The functional integrity of the experimental setup was assessed through three fundamental binary assays: an empty tent versus an empty tent, the standard blend versus the standard blend and person LA versus person HA. Each experimental comparison was carried out for four nights with rotation of stimuli between the tents to avoid positional bias. Two experiments were conducted per night; between 1930 to 2000 hours and 2030 to 2100 hours. Temperature and relative humidity of tents occupied by person LA and person HA were recorded in all replicates using data loggers (Tinytag^®^). The average temperature and relative humidity readings in tents occupied by each individual were calculated.

### Responses to synthetic attractants

Comparative mosquito behavioural responses to human emanations and synthetic attractants were evaluated. The specific binary tests included (i) person LA versus an empty tent, (ii) person LA versus the standard blend, (iii) person LA versus blend IB1, (iv) person LA versus his worn sock, (v) person HA versus an empty tent, (vi) person HA versus the standard blend, (vii) person HA versus blend IB1 and (viii) person HA versus his worn sock. The competing odour sources were placed in separate tents (Figure 1). Each experimental comparison was carried out for four nights. Two experiments were conducted per night; between 1930 to 2000 hours and 2030 to 2100 hours. Odour sources were alternated between tents on each experimental night. Mosquitoes orienting towards odour laden air emanating from a specific tent were recovered from collecting cages present in trap chambers adjacent to that tent, counted and recorded as having been attracted by the material baiting the tent. Natural host odours were obtained from volunteers either by sucking air from tents in which they lay or by using 100% cotton socks worn by them over a period of 8 hours and placed in an MM-X trap. Natural odour collected onto participants' worn socks and synthetic odour in the form of the standard blend or IB1 were dispensed using MM-X traps (American Biophysics Corporation, North Kingstown, RI, USA). To do this, the worn socks or bundles of nylon strips representative of the standard blend or IB1 were placed inside the central tube of an MM-X trap before power (12 V) was applied.

Natural human odours (whole human emanations and foot odours) served as positive controls against which candidate synthetic attractants (standard blend and Ifakara blend 1) were screened.

### Responses to synthetic attractants augmented with heat and moisture

Temperature and/or relative humidity of tents containing the candidate synthetic attractants were raised to the levels pre-determined for tents occupied by person LA or person HA, respectively, depending on whether person LA or person HA participated as the human odour source. The aim was to determine the effect of heat and/or moisture on attraction of mosquitoes to attractants of synthetic (i.e. the standard blend and blend IB1) and natural (worn socks) origin. Thus, the effect of heat on relative attractiveness of person LA was determined by comparing the number of mosquitoes attracted to person LA versus those attracted to the competitor stimuli (worn socks, standard blend or blend IB1) when heated to the average temperature predetermined for a tent occupied by person LA. The effect of heat on relative attractiveness of person HA was determined similarly. Likewise, the effect of moisture on relative attractiveness of person LA or person HA was determined by comparing the number of mosquitoes attracted to person LA or person HA versus those attracted to the competitor stimuli (worn socks, standard blend or blend IB1) when moisturized to the average relative humidity predetermined for a tent occupied by person LA or person HA. Equally, the combined effect of heat and moisture on relative attractiveness of person LA or person HA was determined by comparing the number of mosquitoes attracted to person LA or person HA versus those attracted to the competitor stimuli (worn socks, standard blend or blend IB1) when heated and moistened to the average temperature and relative humidity predetermined for tents occupied by person LA or person HA. The tents were heated and/or humidified using a portable heater (Conrad Electronics^®^) regulated with a thermostat and/or a portable humidifier regulated with an inbuilt humidistat (Honeywell^®^).

### Ethical considerations

The goal, objectives, rationale and procedures of the study were explicitly explained to the human subjects until they understood them clearly. The volunteers' consent to participate in the study was sought thereafter and recruitment in the study done upon consent. This study was approved by the joint Kenyatta National Hospital/University of Nairobi ethical review committee (protocol approval number P102/7/2004 amended in 2008).

### Statistical analysis

The factors that would potentially affect the mosquito responses i.e. tent and experimental period were both categorical, while behavioural responses of individual mosquitoes were mutually exclusive. Conformity of the datasets to a Poisson distribution was authenticated through dispersion tests, which examined whether means equalled the variances [34]. Rigorous statistical analyses were undertaken thereafter. Using Generalized Linear Models [35] the number of mosquitoes attracted to the different sources of behavioural stimuli (human subjects, their worn socks or synthetic attractants) was modelled as a proportion of the total number of mosquitoes recovered from the choice chamber, the release cup and the two trap chambers. The data were transformed to assume a normal distribution using a logarithm link function. The analyses allowed for differences to be determined between odour baits, traps and test periods. A model of the form log (μ_*ijk*_) = E_*i *_+ T_*j *_+ B_*k*_, where E_*i*_, T_*j *_and B_*k *_are the parameter estimates for experimental period *i*, trap *j *and behavioural stimulus *k*, respectively, was fitted. Thus, the proportion of mosquitoes attracted to behavioural stimulus B_*k *_was estimated by the following equation:

The extent to which different pairs of contrasting behavioral stimuli activated or attracted mosquitoes allowed for differences between test periods only. Parameter estimates provided an index of attraction of mosquitoes to the different behavioural stimuli. The symmetry and functional integrity of the experimental setup was assessed using tent A, tent A and person HA as references in three baseline binary assays i.e. empty tent versus empty tent, standard blend versus standard blend and person HA versus person LA, respectively. In all other binary assays (i.e. those assessing relative attraction of humans to synthetic attractants on their own or when augmented with heat, moisture or both) the human subjects posed as baseline references against which the degree of attraction of the mosquitoes to the synthetic attractants was compared. The General statistical software programme (GenStat Discovery Edition 3) was used to analyse the data [36].

## Results

A total of 30 dual-choice tests were conducted over a period of 120 nights. The experiments were carried out between February and November, 2008. A total of 24,000 laboratory reared female *An. gambiae *mosquitoes were used. Mosquito behavioural responses to competing stimuli were assessed within the short-range phase (< 2 metres) of attraction. Two human subjects, neither of whom was found with malaria parasites throughout the study, participated in the experiments.

### Testing functional integrity of the dual-choice setup

No mosquitoes were caught in the trapping chambers when both tents were left empty. Subsequent tests found no significant differences in the number of mosquitoes attracted to the standard blend when competed against itself (P = 0.889). The relative attractiveness of the volunteers differed significantly (P < 0.001). One hundred percent of the mosquitoes were attracted to the person HA. The average temperature and relative humidity in the tents occupied by the less and highly attractive individuals were 24.98°C and 75.28% and 25.5°C and 80.43%, respectively. These three experiments confirmed that the experimental setup was well suited for discriminating mosquito behavioural responses to candidate behavioural stimuli (Table 1).

**Table 1:** Proportions of mosquitoes attracted (reference) in the absence of human emanations or in the presence of synthetic and natural (human-derived) behavioural stimuli in binary assays

Behavioural stimuli		N	P	Reference	Mosquito behavioral responses	
				
Reference	Other				n	Net attraction
Tent A (empty)	Tent B (empty)	8	1.000	0.00	559	0.00^a^
Standard Blend	Standard Blend	8	0.889	0.51	527	0.10^b^
Person HA	Person LA	8	0.001	1.00	541	0.13^b^

N refers to the number of replicates. P values (P) indicate the level of statistical difference between pairs of contrasting stimuli i.e. reference versus other. Proportions of mosquitoes attracted to different reference stimuli (reference) relative to contrasting ones (other) are shown. The total number of mosquitoes collected in the choice and trap chambers (n) and the proportion of these that were attracted to either behavioural stimulus (Net attraction) are shown. Values followed by different letter superscripts in the same column differ significantly.

### Responses to synthetic attractants

A higher proportion of mosquitoes were attracted to the less as well as the highly attractive person used in the study when compared to the standard blend (63% versus 37%, P < 0.001 and 80% versus 19%, P < 0.001, respectively). On the contrary, whereas the proportion of mosquitoes attracted to the less attractive person versus blend IB1 (49% versus 51%, respectively; P = 0.417) or his worn socks did not differ (61% versus 39%, respectively; P = 0.613), far more mosquitoes were attracted to the highly attractive individual in comparison to blend IB1 (96% versus 4%; P = 0.001) or his worn socks (91% versus 9%; P = 0.001). The less as well as the highly attractive individual attracted a significantly higher proportion of mosquitoes compared to empty tents (83% versus 17%, P < 0.001 and 93% versus 7%, P < 0.001, respectively). These results are depicted in Table 2.

**Table 2:** Attraction of *Anopheles gambiae *to humans versus odours of synthetic and natural origin.

Behavioural stimuli		N	P	Person	Mosquito Behavioural Responses	
				
Person	Other				n	Net attraction
LA	Empty tent	8	0.001	83%	511	0.07^a^
LA	Standard Blend	8	0.001	63%	538	0.04^b^
LA	Blend IB1	8	0.417	49%	548	0.14^c^
LA	Worn sock	8	0.613	61%	629	0.16^c^
HA	Empty tent	8	0.001	93%	563	0.28^d^
HA	Standard Blend	8	0.001	80%	444	0.27^d^
HA	Blend IB1	8	0.001	96%	562	0.28^d^
HA	Worn sock	8	0.001	91%	559	0.36^e^

N refers to the number of replicates. P values (P) indicate the level of statistical difference between pairs of contrasting stimuli i.e. person versus competing behavioural stimuli (other). Percentages of mosquitoes attracted to the persons are shown. The total number of mosquitoes collected in the choice and trap chambers (n) and the proportion of these that were attracted to either behavioural stimulus (Net attraction) are shown. Values followed by different letter superscripts in the same column differ significantly.

### Responses to synthetic attractants augmented with heat and moisture

There was no significant difference (P = 0.650) in the proportion of mosquitoes attracted to person LA (52%) when compared to his worn socks (48%) augmented with heat (24.98°C). However, person LA attracted a significantly higher proportion of mosquitoes when compared to the standard blend (73% versus 27%, P < 0.001) and blend IB1 (61% versus 39%, P = 0.016) when each was augmented with heat (24.98°C) (Table 3). Person LA attracted a significantly higher proportion of mosquitoes when compared to the standard blend (99% versus 1%, P < 0.001), blend IB1 (98% versus 2%, P < 0.001) or his worn socks (98% versus 2%, P < 0.001) when each was augmented with moisture (75.28%) (Table 4). Although person LA attracted a significantly higher proportion of mosquitoes compared to the standard blend (100% versus 0%, P < 0.001) and his worn socks (80% versus 20%, P < 0.001) when each was augmented with heat (24.98°C) and moisture (75.28%), there was no difference in the proportion of mosquitoes attracted to him when compared to blend IB1 (41% versus 59%, P = 0.416) augmented with heat (24.98°C) and moisture (75.28%) (Table 4).

**Table 3:** Attraction of *Anopheles gambiae *to humans versus odours of synthetic and natural origin augmented with heat.

Behavioural stimuli		N	P	Person	Mosquito behavioral responses	
				
Person	Other				n	Net attraction
LA	Standard Blend + Heat	8	0.001	73%	553	0.18^a^
LA	Blend IB1 + Heat	8	0.016	61%	505	0.21^a^
LA	Worn sock + Heat	8	0.650	52%	579	0.13^b^
HA	Standard Blend + Heat	8	0.001	100%	651	0.33^c^
HA	Blend IB1 + Heat	8	0.001	99%	568	0.31^c^
HA	Worn sock + Heat	8	0.001	97%	595	0.19^a^

N refers to the number of replicates. P values (P) indicate the level of statistical difference between pairs of contrasting stimuli i.e. person versus competing behavioural stimuli (other). Percentages of mosquitoes attracted to the persons are shown. The total number of mosquitoes collected in the choice and trap chambers (n) and the proportion of these that were attracted to either behavioural stimulus (Net attraction) are shown. Values followed by different letter superscripts in the same column differ significantly.

**Table 4:** Attraction of *Anopheles gambiae *to humans versus odours of synthetic and natural origin augmented with moisture.

Behavioural stimuli		N	P	Person	Mosquito behavioral responses	
				
Person	Other				n	Net attraction
LA	Standard Blend + Moisture	8	0.001	99%	663	0.21^ab^
LA	Blend IB1 + Moisture	8	0.001	98%	627	0.22^a^
LA	Worn sock + Moisture	8	0.001	98%	589	0.18^b^
HA	Standard Blend + Moisture	8	0.001	100%	611	0.28^c^
HA	Blend IB1 + Moisture	8	0.001	100%	643	0.24^ac^
HA	Worn sock + Moisture	8	0.001	99%	551	0.28^c^

N refers to the number of replicates. P values (P) indicate the level of statistical difference between pairs of contrasting stimuli i.e. person versus competing behavioural stimuli (other). Percentages of mosquitoes attracted to the persons are shown. The total number of mosquitoes collected in the choice and trap chambers (n) and the proportion of these that were attracted to either behavioural stimulus (Net attraction) are shown. Values followed by different letter superscripts in the same column differ significantly.

Person HA attracted a significantly higher proportion of mosquitoes when compared to the standard blend (100% versus 0%, P < 0.001), blend IB1 (99% versus 1%, P < 0.001) or his worn socks (97% versus 3%, P < 0.001) when each was augmented with heat (25.5°C) (Table 3). He similarly attracted a significantly higher proportion of mosquitoes when compared to the standard blend (100% versus 0%, P < 0.001), blend IB1 (100% versus 0%, P < 0.001) or his worn socks (99% versus 1%, P < 0.001) when each was augmented with moisture (80.43%) (Table 4). As well, person HA attracted a significantly higher proportion of mosquitoes when compared to the standard blend (98% versus 2%, P < 0.001), blend IB1 (99% versus 1%, P < 0.001) or his worn socks (100% versus 0%, P < 0.001) when each was augmented with heat (25.5°C) and moisture (80.43%) (Table 5).

**Table 5:** Attraction of *Anopheles gambiae *to humans versus odours of synthetic and natural origin augmented with heat plus moisture.

Behavioural stimuli		N	P	Person	Mosquito behavioral responses	
				
Person	Other				n	Net attraction
LA	Standard Blend + Heat + Moisture	8	0.001	100%	539	0.19^a^
LA	Blend IB1 + Heat + Moisture	8	0.416	41%	634	0.04^b^
LA	Worn sock + Heat + Moisture	8	0.001	80%	614	0.11^c^
HA	Standard Blend + Heat + Moisture	8	0.001	98%	673	0.13^c^
HA	Blend IB1 + Heat + Moisture	8	0.001	99%	669	0.25^d^
HA	Worn sock + Heat + Moisture	8	0.001	100%	631	0.08^e^

N refers to the number of replicates. P values (P) indicate the level of statistical difference between pairs of contrasting stimuli i.e. person versus competing behavioural stimuli (other). Percentages of mosquitoes attracted to the persons are shown. The total number of mosquitoes collected in the choice and trap chambers (n) and the proportion of these that were attracted to either behavioural stimulus (Net attraction) are shown. Values followed by different letter superscripts in the same column differ significantly.

## Discussion

This study demonstrates the importance of olfactory cues in mediating the host-seeking behaviour of *Anopheles gambiae *under semi-field conditions. Since evaluations were based on mosquito behavioural responses at short range [33,37], the results cannot be extrapolated to explain events that occur in the medium and long-range phases of attraction. The apparent lability between the behaviour of field and laboratory mosquito populations [38] also restricts interpretation of the results to the semi-field situations under which experiments were conducted. Inherent differences in human attractiveness to mosquitoes [29,33,39,40] were seen to affect the efficacy of synthetic attractant blends in attracting the mosquitoes. This calls for caution whenever human subjects participate as volunteers in field efficacy, effectiveness and epidemiological studies.

The high proportion of mosquitoes caught in the trapping chambers in the absence of human cues i.e. by empty tents is hard to explain. Two hypotheses are construed. The first is that moving air is an activator for *An. gambiae*. The second is that *An. gambiae*, having evolved with humans and being highly anthropophilic [41], exhibits behavioural responses not only to human-derived chemical stimuli but also to behavioural stimuli originating from items commonly found in human dwellings and in the intra domiciliary environment. It is noteworthy that no mosquitoes were attracted to any of the tents when both were empty. This, coupled with the fact that there were no differences in the proportion of mosquitoes trapped when the two tents were baited with the same stimulus i.e. standard blend confirms that the behavioural responses were symmetrical. That differences in relative attractiveness between two persons could be discerned was not surprising. This is in conformity with previous studies where a similar discriminatory system was used [33,37,42].

Each of the two volunteers, one less and the other highly attractive to *An. gambiae *[33], attracted a higher proportion of mosquitoes when compared to empty tents or the standard blend. *Anopheles gambiae *prefers to feed on humans rather than other animal hosts [1,2,43]. Thus, it is not surprising that relatively more mosquitoes were attracted by the humans. This finding concurs with those reported by Mboera *et al *[44] and Mukabana *et al *[37], although evolutionary plasticity in the behaviour of field and laboratory populations exists [38]. The standard blend used in this study contained carbon dioxide (released at 500 ml/min), ammonia (2.5%) and distilled water. Failure of this blend to attract more or as many mosquitoes as a human may be attributed to (i) its limited chemical composition relative to the large diversity of compounds emanating from humans [45,46], (ii) lack of constituents active in the short range phase of attraction as limited by the experimental setup [33], and (iii) lack of compounds distinctive and specific of human odour [47].

Whereas the proportion of mosquitoes attracted to the less attractive person versus Ifakara blend 1 (IB1) or his worn socks did not differ; far more mosquitoes were attracted to the highly attractive person relative to IB1 and his worn socks. Furthermore, the total proportion of mosquitoes attracted were low and high when the less and highly attractive persons were used as controls, respectively. This was irrespective of the contrasting behavioural stimuli against which they were compared and whether the stimuli were augmented with heat, moisture or both. These findings suggest that the inherent differences in peoples' attractiveness to host-seeking mosquitoes [29,33,39,40] undermine the use of human subjects as screens for potential mosquito attractants [32]. It, therefore, matters the identity of the specific human subject against whom candidate mosquito attractants are screened. A standard, highly stringent positive control for screening candidate mosquito attractants should be sought. A good positive control, while closely depicting the key 'essence-of-man', should maintain a high and relatively unchanged degree of attraction to host-seeking mosquitoes under standardized climatic conditions.

Lack of statistical difference in the proportion of mosquitoes attracted to the less attractive person versus his worn socks indicates that the degree of attractiveness to mosquitoes is dictated by foot odours. This corroborates previous findings in which *An. gambiae *was shown to have a distinct preference for biting the legs and feet [48,49]. Several authors have since demonstrated attraction of *An. gambiae *to human foot odours [24,26,27]. However, because total body emanations of the highly attractive person attracted far more mosquitoes than his worn socks suggests that sources of attractive odours abound in other human body parts. Indeed, there are several reports of attraction of *An. gambiae *to material obtained from body parts other than the feet [29,50,51].

The highly attractive individual attracted far more mosquitoes (≥ 97%) than the standard blend, blend IB1 or his worn socks when these behavioural stimuli were augmented with heat, moisture or both. If both physical and chemical cues are responsible for attracting host-seeking mosquitoes to humans [5,52,53] then it is plausible to infer that behavioural stimuli emanating from the highly attractive individual had combinations of both cues. The complimentary thinking is that the critical chemical compounds responsible for attracting host-seeking mosquitoes to humans were not present in the contrasting behavioural stimuli. This reasoning partially applies to data associated with the less attractive individual who was more attractive when the three behavioural stimuli were augmented with moisture alone or when the standard blend was augmented with heat, moisture or both.

Augmenting blend IB1 with heat or moisture did not increase its attractiveness over the less attractive individual, however, adding heat plus moisture rendered these contrasting sources of behavioural stimuli equally attractive to *An. gambiae*. This implies, in concurrence with published literature, that heat and moisture have a synergistic effect on the attraction of mosquitoes to odour baits [6,54,55]. Furthermore, the less attractive individual remained as attractive as his worn socks augmented with heat, but more attractive against his worn socks augmented either with moisture alone or with heat plus moisture. This finding underscores similarities in the behaviour of *An. gambiae *and other mosquito species. Even though heat may act synergistically with odour baits to increase their attraction to mosquitoes [28,55-58], this phenomenon is, with few exceptions [6], overridden in the presence of moisture, which reduces attraction of mosquitoes to odour baits [54]. Mosquitoes used in this study were starved for eight hours during which period they were supplied with water on cotton wicks. Water-satiated mosquitoes, unlike thirsty counterparts, are known to avoid environments with high relative humidity [54]. This potentially explains why the volunteers consistently attracted more mosquitoes over odour baits augmented with moisture. An exception to this was recorded when the less attractive person was compared to blend IB1 augmented with heat plus moisture.

Failure of heat and moisture to change the relative attractiveness of mosquitoes to the candidate odour baits can be attributed to temperature and relative humidity fluctuations i.e. between 25-27°C and 75-85%, respectively. As small differences in the levels of these parameters can associate with major differences in mosquito behavioural responses [59], this may have been the case in our experiments. *Anopheles gambiae*, being highly anthropophlic [1,2,43], has evolved to respond to human-specific rather than generalist cues. Ideally, heat and moisture, being host-unspecific, should, in interaction with other host-specific cues at short range [6,41], be indicative of the physical presence of hosts and thus important in inducing landing rather than act as cues mediating directional responses on their own. In our experimental setup attracted mosquitoes were trapped while flying upwind towards the odour baits, so excluding the behavioural end point of landing.

Of the three sources of behavioural stimuli tested, Blend IB1 emerged as the most potent mosquito attractant. The proportion of mosquitoes attracted to it equalled those attracted to the less attractive human subject when the blend was used on its own or when it was augmented with heat plus moisture. Except in one case [26], the key components of blend IB1 have been shown to be highly attractive to mosquitoes under laboratory [12,13], semi-field [15] and field conditions [14,15]. The fact that blend IB1 was consistently less attractive than the highly attractive human subject suggests that more compounds need to be added to blend IB1 and the concentrations of the existing ones refined.

## Conclusion

This study, in concordance with existing literature [5,8,52,53,60,61], supports previous reports that olfactory cues are the important signals mediating mosquito host location. Heat and moisture enhanced attraction of mosquitoes to candidate odour baits but did not influence the relative attractiveness of the odour baits. That results of attraction of the mosquitoes to the different odour blends varied depending upon the human subject against which they were screened implies that a standard, highly stringent positive control should be sought. Only then can we compare data across different ecological zones, mosquito populations, seasons and experimental setups (i.e. under laboratory, field and semi-field conditions) effectively.

## Competing interests

The authors declare that they have no competing interests.

## Authors' contributions

WRM conceived of the study and designed experiments. WRM designed the experimental setup. EAO carried out experiments with the assistance of MNO. Data analysis was done by EAO together with MNO and WRM. WRM interpreted the data assisted by EAO, MNO, PAM and EDK. WRM wrote the manuscript assisted by EAO. All authors revised the manuscript.
